# The impact of epigenetic modifications on allogeneic hematopoietic stem cell transplantation

**DOI:** 10.3389/fimmu.2023.1188853

**Published:** 2023-05-31

**Authors:** Yiouli P. Ktena, Margarita Dionysiou, Lukasz P. Gondek, Kenneth R. Cooke

**Affiliations:** Department of Oncology, Sidney Kimmel Comprehensive Cancer Center, Johns Hopkins University School of Medicine, Baltimore, MD, United States

**Keywords:** bone marrow transplant (BMT), allogenetic hematopoietic stem cell transplantation, DNA methylation, epigenetics, GvHD, histone modifications, non-coding RNA

## Abstract

The field of epigenetics studies the complex processes that regulate gene expression without altering the DNA sequence itself. It is well established that epigenetic modifications are crucial to cellular homeostasis and differentiation and play a vital role in hematopoiesis and immunity. Epigenetic marks can be mitotically and/or meiotically heritable upon cell division, forming the basis of cellular memory, and have the potential to be reversed between cellular fate transitions. Hence, over the past decade, there has been increasing interest in the role that epigenetic modifications may have on the outcomes of allogeneic hematopoietic transplantation and growing enthusiasm in the therapeutic potential these pathways may hold. In this brief review, we provide a basic overview of the types of epigenetic modifications and their biological functions, summarizing the current literature with a focus on hematopoiesis and immunity specifically in the context of allogeneic hematopoietic stem cell transplantation.

## Introduction

Every cell in the human body carries the same genetic code, yet only a subset of genes is actively expressed at any given time-point in any given cell, in an intricate process that is orchestrated by epigenetics. In the Greek language, “epi” signifies on, upon, or over. Accordingly, the field of epigenetics studies the processes that affect gene expression without altering the DNA sequence itself, the sum of which is described as the epigenome (1, 2).

Epigenetic modifications include DNA methylation, histone modification, Chromatin remodeling, and non-coding RNA regulation (1–3). In brief, a nucleosome, chromatin’s basic structural unit, is comprised of negatively charged DNA packed around a positively charged histone octamer. Chemical alterations to this complex can allow or prevent access of the transcriptional machinery to the DNA sequence. Broadly, epigenetic regulators may be classified as “writers”, “readers”, and “erasers”. Writers include a wide variety of enzymes that introduce modifications on DNA and histones, including DNA methyltransferases, histone methyltransferases, histone acetyltransferases. Readers encompass proteins with domains that recognize and bind specific epigenetic modifications, such as methyl-CpG-binding proteins, histone methylation binding proteins, and histone acetylation binding proteins. Finally, erasers represent proteins which actively remove epigenetic marks and reverse the effects on transcription, such as TET (ten-eleven translocation) proteins which catalyze cytosine demethylation, histone demethylases, and histone deacetylases (4).

Many of these changes can be mitotically and/or meiotically heritable on nascent daughter chromatin strands upon cell division. This results in a type of cellular memory termed epigenetic memory. At the same time, epigenetic modifications are characterized by plasticity in response to factors intrinsic and extrinsic to the cell, such as environmental stimuli (5–7). Therefore, not only can these modifications somatically be inherited after cell division and repress target gene transcription, but they can also be reversed during transitions between cellular fates, making them the focus of significant scientific investigation.

## Mechanisms of epigenetic modification

DNA methylation is the most widely studied form of epigenetic modification and generally results in gene silencing. It primarily involves methylation of cytosine residues almost exclusively in the context of CpG dinucleotides. CpG dinucleotides cluster in regions termed CpG islands, where approximately 60% of human gene promoters are located. However, tissue-specific DNA methylation and differential methylation associated with reprogramming are mostly located in regions adjacent to each side of a CpG island, termed shores (1–3). Of the five known human DNA methyltransferases, DNMT1 is thought to be primarily responsible for maintenance methylation, while DNMT3A and DNMT3B are the primary *de novo* methyltransferases. DNMT3L lacks enzymatic activity but interacts with other enzymes and plays a role in maternal imprinting (8, 9).

Histone modifications, at various sites, include methylation (which most commonly represses transcription), acetylation (usually activating in nature), phosphorylation (which contributes to chromatin remodeling and assists in DNA repair), ubiquitylation, and others (1–3, 7). These modifications, along with histone variants within nucleosomes (histones characterized by minor differences in their amino acid sequence from their canonical counterpart) can also result in nucleosome repositioning and chromatic remodeling, such that transcription sites are more or less accessible to interact with transcriptional machinery (1, 3). These chromatin markers and the associated epigenetic signatures have shown to be reversible (1).

Lastly, non-coding RNAs (ncRNAs), particularly regulatory ncRNAs, including microRNAs (miRNAs), long non-coding RNAs (lncRNAs), circular RNAs (circRNAs), small interfering RNAs (siRNAs) and piwi-interacting RNAs (piRNAs), are transcribed (but non-translated) RNA molecules believed to fine-tune gene expression by contributing to gene silencing. Moreover, non-coding RNAs are known to interact with and guide chromatin-modifying complexes to the appropriate genomic targets (1–3, 5, 7). Notably, the aforementioned epigenetic mechanisms are functionally linked and interdependent, with continuous cross-talk involving both positive and negative feedback.

## Epigenetics in human development, health, and disease

Epigenetic processes appear to have an instrumental role in human development, health, and disease (7, 10). In the earliest stages of embryogenesis, the epigenome is “reset”. As the zygote, in its one cell glory, transitions from totipotency to pluripotency in the blastocyst stage, complex epigenetically controlled mechanisms guide each cell towards a differentiated state and limit plasticity to maintain a fixed cell type (11, 12). Highlighting the importance of epigenetics in embryogenesis is the fact that *Dnmt3a-* and/or *Dnmt3b*-null mice die during gestation or shortly after birth (13).

As suggested, epigenetic dysregulation can disrupt cellular homeostasis and has been implicated in various diseases (6, 7, 14, 15). Disorders affecting genomic imprinting can result in distinct clinical syndromes, such as Beckwith-Wiedemann, Russell-Silver, and Prader-Willi/Angelman syndromes (16), while germline *DNMT3A* mutations can result in Tatton-Brown-Rahman syndrome (17, 18). In the early eighties, global DNA hypomethylation was first described in cancer. Subsequent work revealed that focal DNA hypermethylation of tumor suppressor genes facilitates stem-like cell behavior in the pathophysiology of carcinogenesis (19–22). Mutations in *DNMT3A* (the product of which catalyzes *de novo* DNA methylation), *TET2* (responsible for regulated demethylation), and *ASXL1* (involved in chromatin regulation), are the most frequently described mutations in clonal hematopoiesis, including clonal hematopoiesis of indeterminate potential (CHIP) and myeloid malignancies such as myeloproliferative neoplasms (MPN), myelodysplastic syndrome (MDS), and acute myeloid leukemia (AML) (23–25). CHIP has been recently identified as a new precursor state for myeloid malignancies (26, 27). Specific types of histone modifications and microRNAs have also been linked to a multitude of malignancies (28–31). In this context, an ever-increasing number of epigenetically targeted therapies have been developed and applied in the care of cancer patients, whether alone or in combination with traditional chemotherapy, targeted agents, or immunotherapy (32, 33). Epigenetic mechanisms with therapeutic potential have also been implicated in neurodegenerative disorders (3, 34).

## Epigenetic regulation of hematopoietic stem cells, before and after transplantation

Hematopoietic stem cells (HSCs) are characterized by self-renewal and pluripotency, features that are of paramount importance to the biological functions of hematopoiesis and immunity. As multipotent progenitors arise from HSCs and further commit to the myeloid or lymphoid lineage, epigenetic regulators define the major differentiation and maintenance events (14). In particular, DNA methylation plays a crucial role in these processes. Epigenetic factors are also associated with cellular senescence, the inevitable cessation in proliferation that characterizes biological aging. In HSCs, cellular aging is associated with a relative increase in the myeloid component, enhanced autoimmunity, accrual of DNA damage, and an increased risk of hematologic neoplasms (35). Genome-wide studies of HSCs show distinct differences in the epigenomic landscape of aging cells with several regions of differential DNA methylation having potential relevance for age-related disease (36). *Dnmt1* deletion in murine HSCs results in rapid death from profound pancytopenia due to absence of all HSCs and progenitors, while low-level *Dnmt1* expression results in a marked decrease of lymphoid progenitors and decreased self-renewal capacity in serial transplantation experiments (37). *Dnmt3a/Dnmt3b*-deficiency in murine HSCs leads to a decline in their differentiation potential with accumulation of hematopoietic progenitors in the marrow. Furthermore, *Dnmt3a*-null and *Tet2*-null cells have a competitive expansion and survival advantage in serial transplantation experiments (38–43). ASXL1 proteins are epigenetic regulators that recruit chromatin modification complexes and transcription factors; in mice, *Asxl1* deficiency in HSCs results in myelodysplasia with accumulation of hematopoietic progenitors but decreased self-renewal, and serial transplantation of *Asxl1*-null HSCs results in acceleration of a lethal myelodysplastic disorder as compared to primary *Asxl1* KO mice (44). *Tet2*-deficient mice demonstrate increase hematopoietic self-renewal and compound *Asxl1* and *Tet2* loss restores the *Asxl1*-loss related self-renewal defect and results in more severe MDS-like features. These findings are in accordance with the fact that *DNMT3A*, *TET2*, and *ASXL1* mutations are some of the most common genetic abnormalities in clonal hematopoiesis and are frequently detected, alone or in combination, in patients with hematological malignancies (23, 45–48).

In the post-transplant setting, allogeneic hematopoietic stem cell transplantation (HSCT) recipients have been found to stably maintain the donor’s global methylation status, and differences in global methylation correlate with the evolution of mixed chimerism (49). Moreover, donor methylation levels in the promoters of critical genes such as *IFNG* and *FASL* correlate with the severity of acute graft-versus-host disease (GVHD), suggesting they could be used alongside HLA typing to optimize donor selection (49). Apart from specific DNA methylation signatures, the “epigenetic age” of donor HSCs appears to be cell-intrinsic, and thus remains largely stable after transplantation in concordance with the chronological age of the donor. As such, epigenetic age is not influenced by the recipient’s chronological age, even decades after transplantation (50–52). In two separate studies, patients whose donor stem cells exhibited accelerated aging, as determined by the “epigenetic clock”, were at higher risk of developing chronic GVHD (53, 54). In xenogeneic murine models of HSCT, this phenomenon is again thought to be cell-intrinsic, rather than host-dependent (55).

Importantly, several studies have now linked donor CHIP to HSCT outcomes. A European study identified 92 clonal mutations in 500 healthy donors over the age of 55; donor CHIP, especially *DNMT3A*-driven CHIP, was associated with increased incidence of chronic GVHD and decreased incidence of relapse or progression. Subsequently, a large US-based study of over 1,700 donors over the age of 40 further confirmed these findings. Additional data suggest that donor *DNMT3A* mutations are independently associated with improved overall survival due to reduced risk of relapse (56–58). Of note, this phenomenon appears to be eliminated by the administration of post-transplantation cyclophosphamide for the prevention of GVHD, suggesting that it is at least partially mediated by *DNMT3A*-mutated donor T-cells, the function of which is altered by cyclophosphamide (58, 59). In these studies, there was a very low risk of donor CHIP evolution to donor cell leukemia (DCL): 2 recipients of 82 mutated grafts in the first study and 6 recipients of 388 mutated grafts in the second (56, 58, 60). No recipients with sole mutations in *DNMT3A* or *TET2* developed DCL. Two smaller studies also examined the CHIP-alloreactivity link: A single-center study found increased risk of acute GVHD but not chronic GVHD and no differences in incidence of relapse; notably, this study included a large number of high-risk patients, with over half of the cohort having active disease at the time of transplantation (61). A more recent study also failed to replicate the results from the larger studies, likely due to the significantly limited sample size (only 25 mutated donor products were identified, with an unusual distribution of CHIP mutations, potentially due to thefact that donors as young as 17 were included) (62). GVHD following liver transplantation (LT-GVHD) is a rare complication, associated with bone marrow failure and a hyperinflammatory state; in a case series of 9 patients where 7 bone marrow samples were available for next generation sequencing, *DNMT3A* mutations were found in 5 out of 7 samples, as compared to 1 of 6 in a LT-non-GVHD cohort (63).

In accord with these clinical observations, laboratory data showed an increase in both acute and chronic GVHD when T-cell lineage-specific *Dnmt3a*-null mice were used as donors in multiple murine allogeneic HSCT models (58, 64). These observations were associated with early proliferation of donor-derived *Dnmt3a*-null T-cells as compared to wild-type T-cells. Furthermore, *Dnmt3a*-null T-cells demonstrated a migration advantage to the gastrointestinal tract and secondary lymphoid organs, enhanced pro-inflammatory cytokine production, and decreased expression of exhaustion and apoptosis markers (64). A comprehensive review of the epigenome and transcriptome of *Dnmt3a*-null donor T-cell subsets post-HSCT, via whole genome bisulfite sequencing and in parallel, bulk RNA sequencing, showed similar global DNA methylation levels to wild-type T-cells but distinct hypomethylation peaks in gene pathways involved with T-cell activation and differentiation. More importantly, donor T-cells lacking DNMT3A provided superior tumor control in graft-versus-leukemia models, corroborating the clinical data cited above (64). In gene-set enrichment analyses of the genes differentially expressed between *Dnmt3a-*null and WT donor T-cells, CD8+ T-cells lacking DNMT3A were highly enriched for effector-like signatures and negatively enriched for exhaustion-like signatures, while CD4+ T-cells were enriched for genes expressed in activated and progenitor cell populations.

These observations are consistent with a growing body of data showing that DNA methylation critically contributes to the functional properties that define T-cell identity. For example, DNMT3A is selectively upregulated 38-fold following T-cell receptor stimulation and subsequently regulates T-helper cell polarization and effector T-cell differentiation, depending on the context of cellular activation (65–67). Following differentiation and activation, the patterns of gene expression that define T-cell subsets are stabilized through DNA methylation. T-cell deletion of DNMT3A enhances the plasticity of T-helper cells by allowing for reprogramming of cytokine expression, and allows CD8+ T-cells to overcome epigenetically-defined exhaustion programs in order to more effectively clear chronic infections (65–71). DNMT3A deletion also appears to enhance the anti-tumor effects of T-cells whether in the context of immune-checkpoint inhibition, chimeric-antigen receptor (CAR) T-cells, or allogeneic HSCT (64, 71, 72). Akin to what was observed in murine models of allo-HSCT, deletion of DNMT3A in CAR T-cells resulted in exhaustion-resistant cells with preservation of the cells’ proliferative capacity and ongoing anti-tumor response despite prolonged tumor exposure (72, 73).

Since epigenetic dysregulation is common in neoplasia, and given the reversible nature of epigenetic alterations, pharmacologic agents targeting epigenetic regulators are now regularly used in the treatment of cancer patients (32). Azacitidine and decitabine, the two most widely used hypomethylating agents, are nucleoside analogs that bind and inhibit DNA methyltransferases after being incorporated into newly formed DNA. Both drugs have received FDA approval for the treatment of MDS, AML, and chronic myelomonocytic leukemia. However, both agents are non-specific and despite not being categorized as traditional chemotherapy do have cytotoxic potential, giving rise to adverse events secondary to myelosuppression (32). In addition to their anti-tumor effects, the immune-modulatory potential of azacitidine and decitabine is appealing both in the pre- and post-transplantation setting. Prior to HSCT, these agents are used alone or in combination to decrease disease burden and serve as a bridge to transplant, especially in older patients with MDS and AML (74–77). In the post-transplantation period, there is growing interest in strategies that will help prevent relapse, which is a major driver of mortality (77–79). Preclinical and clinical data suggested that azacitidine may mitigate GVHD without compromising graft-versus-leukemia (GVL) activity (80–82). However, a phase III randomized clinical trial of azacitidine maintenance versus observation post-HSCT for high-risk AML/MDS patients failed to improve relapse-free survival (79, 83). Low-dose decitabine may be more promising, whether alone or in combination with other agents (84, 85). Next-generation DNMT inhibitors, such as guadecitabine, appear to have an improved toxicity profile, and non-nucleoside inhibitors, which have the potential to be more potent and selective, are in development (32, 86).

Histone modifications have been widely targeted in the context of neoplasia and allogeneic HSCT, with several agents inhibiting histone deacetylases now commercially available including but not limited to vorinostat, panobinostat, belinostat, and romidepsin (28, 87, 88). Histone modifications contribute to the regulation of proliferation and cytotoxicity in activated T-cells and histone acetylation was one of the first epigenetic modifications to be studied in preclinical models of acute GVHD (87). Histone deacetylase (HDAC) inhibitors were found to decrease the allo-stimulatory function of dendritic cells, a group of potent antigen-presenting cells known to be instrumental in the induction of acute GVHD, as well as enhance natural regulatory T-cell function, and hence reduce GVHD while preserving GVL effects (89–92). As a result, one of these agents, suberoylanilide hydroxamic acid (SAHA, now known as vorinostat) was tested in two phase I/II trials (NCT00810602, NCT01790568; a third, NCT03842696, underway) for the prevention of GVHD in combination with standard therapy and was found to be safe and potentially effective (93, 94). Panobinostat also showed promising results in two phase I/II studies (NCT01111526, NCT02588339) (95, 96). Results from a phase III trial of panobinostat as post-HSCT maintenance therapy are pending (NCT04326764) (79). Enhancer of zeste homolog 2 (EZH2) is a histone methyltransferase that catalyzes histone 3 lysine 27 trimethylation, a modification that represses gene transcription and is thought to be involved in T-cell immune responses (87). Pharmacologic inhibition of histone methylation via DZNep, an inhibitor that depletes EZH2, was found in one study to arrest ongoing GVHD in mice by inducing apoptosis of alloreactive T-cells without inhibiting donor-derived hematopoiesis or GVL activity (97, 98). However, these findings were not replicated with other agents or in a xenogeneic model using DZNep (99).

Bromodomain and extraterminal (BET) proteins regulate chromatin dynamics by binding acetylated lysine residues in histones and nonhistone proteins, including transcription factors; given the therapeutic potential, BET inhibitors targeting the acetyl-binding domains of these proteins have been developed (100). In murine models of GVHD, BET inhibition suppressed GVHD by altering the cytokine expression profiles of dendritic cells and T-cells, with retention of anti-tumor effects, while certain inhibitors also allow for infused Treg expansion as a combinatorial strategy (101, 102). More recently, Snyder et al. reported on two potent and selective BET inhibitors which improve survival and reduce GVHD severity in mice without sacrificing the beneficial GVL effect; PLX51107 is currently being tested in a phase I/II trial for steroid-refractory acute GVHD (NCT04910152) (103). Both EZH2 inhibition and BET-bromodomain inhibition demonstrated activity in preclinical models of chronic GVHD with lung involvement, with evidence of altered transcriptomes in the germinal centers of treated animals (104).

The role of non-coding RNAs, mainly microRNAs, in T-cell immunobiology is well established and is increasingly being explored in the context of hematopoiesis and allogeneic HSCT (105, 106). Several microRNAs are emerging as important regulators of allogeneic T-cells and may prove to be highly sensitive and specific biomarkers for GVHD and useful targets for anti-GVHD oligonucleotide-based therapeutics (105–108). Ranganathan et al., showed increased expression of microRNA-155 (miR-155) in both CD4- and CD8-positive cells following murine allo-HSCT, as well as ameliorated GVHD in multiple KO and antagonist-treated models and worse phenotype in over-expression models (109). MicroRNA-155 also influences GVHD via its function in recipient dendritic cells. In study by Chen et al., miR-155–deficient dendritic cells cause less severe GVHD through reduced migration and defective inflammasome activation, supported by the fact that *Nlrp3/miR-155* double-knockout allo-HSCT recipient mice had no increased protection from GVHD compared with *Nlrp3−/−* recipients (110). The role of the microRNA-17-92 cluster in allo-HSCT was explored in a similar study that utilized a T cell-specific KO model, and showed reduced GVHD by demonstrating defects in proliferation, cytokine production and α4β7 integrin expression in KO T cells (111). MicroRNA-146a has a protective role in GVHD, and its anti-GVHD effects were demonstrated in several studies, via its function in both allogeneic T-cells and recipient dendritic cells (112–114). MicroRNA-31 promotes murine chronic GVHD via T-cell metabolic pathway regulation (108). Differential microRNA expression has been detected in skin biopsies of patients at the time of onset of cutaneous acute GVHD, and circulating microRNAs encapsulated within extracellular vesicles were found differentially expressed in patients with chronic GVHD (115, 116). Several long non-coding RNAs (lncRNAs) have been found to influence the function of T-cells, but their role in allo-immune responses is still unknown. *Linc00402* was recently identified as a long non-coding RNA that regulates T-cell function in humans and experimental murine models. RNA sequencing was performed on human T-cells after HSCT and solid organ (cardiac) transplant and compared to T-cells from healthy subjects. *Linc00402*, a T-cell specific molecule, was found to be differentially expressed in recipients of allogeneic mismatched unrelated as compared to autologous HSCT patients, and in donor T-cells from patients who underwent cardiac transplantation. In contrast, *in vitro* and murine *in vivo* data showed that T-cell activation and proliferation are inversely related to *Linc00402* expression, and that depletion of *Linc00402* impairs the allogeneic stimulation of T-cells *ex vivo*. The authors hypothesized that higher levels of tacrolimus exposure were the culprit for the preservation of *Linc00402* abundance in allogeneic HLA-mismatched HSCT. Importantly, the tissue-specific and allogeneic context-specific expression of this molecule, along with its immune regulatory properties, make for an appealing therapeutic candidate (117, 118). In a study by Wang et.al, numerous lncRNAs were found to be dysregulated in B cells from patients with chronic graft-versus-host disease (cGVHD) as compared to normal counterparts. Specifically, lncRNAs NONHSAT040475, NONHSAT142151 and FR118417 were found to be strongly associated with the BCR signaling pathway in cGVHD pathogenesis (119). Another class of non-coding RNAs, termed circular RNAs, has been associated with increased relapse risk in AML patients (120).

In sum, the maintenance and differentiation of hematopoietic stem cells require epigenetic regulatory networks that are 1) simultaneously heritable and reversible, 2) responsive to both intrinsic and extrinsic stimuli, and 3) characterized by interdependence between the DNA sequence, transcriptional processes, and the various forms of epigenetic changes. Despite the evident importance of epigenetic modifications in hematopoiesis and alloreactivity, the exact mechanistic underpinnings of how epigenetics operate in each context continue to elude the scientific community. Future challenges exist not merely in uncovering the full spectrum of epigenetic circuits, but equally importantly, in defining exactly how these complex, interactive, and frequently conflicting pathways ultimately affect donor stem cells, immune cell reactivity, and HSCT outcomes. As the field of epigenetics continues to steadily evolve, HSCT patients will undoubtedly benefit from the knowledge that is yet to be fully appreciated.

## Author contributions

Conception: YK and KC. Literature review: YK, MD, LG, and KC. Manuscript writing: YK, MD, LG, and KC. All authors contributed to the article and approved the submitted version.

## References

[1] Allis CD Jenuwein T. The molecular hallmarks of epigenetic control. Nat Rev Genet (2016) 17(8):487–500. doi: 10.1038/nrg.2016.59 27346641

[2] Al Aboud NM Tupper C Jialal I. Genetics, epigenetic mechanism. In: StatPearls. Treasure Island (FL): StatPearls Publishing LLC (2022). 30422591

[3] Portela A Esteller M. Epigenetic modifications and human disease. Nat Biotechnol (2010) 28(10):1057–68. doi: 10.1038/nbt.1685 20944598

[4] Nicholson TB Veland N Chen T. Writers, readers, and erasers of epigenetic marks. In: Epigenetic cancer therapy. Elsevier Inc (2015). p. 31–66. Available at: https://www.elsevier.com/books/epigenetic-cancer-therapy/gray/978-0-323-91367-6.

[5] D’Urso A Brickner JH. Mechanisms of epigenetic memory. Trends Genet (2014) 30(6):230–6. doi: 10.1016/j.tig.2014.04.004 PMC4072033 24780085

[6] Feinberg AP. The key role of epigenetics in human disease prevention and mitigation. N Engl J Med (2018) 378(14):1323–34. doi: 10.1056/NEJMra1402513 PMC11567374 29617578

[7] Zhang L Lu Q Chang C. Epigenetics in health and disease. Adv Exp Med Biol (2020) 1253:3-55. doi: 10.1007/978-981-15-3449-2_1 32445090

[8] Hata K Okano M Lei H Li E. Dnmt3L cooperates with the Dnmt3 family of de novo DNA methyltransferases to establish maternal imprints in mice. Development (2002) 129(8):1983–93. doi: 10.1242/dev.129.8.1983 11934864

[9] Lyko F. The DNA methyltransferase family: a versatile toolkit for epigenetic regulation. Nat Rev Genet (2018) 19(2):81–92. doi: 10.1038/nrg.2017.80 29033456

[10] Kanherkar RR Bhatia-Dey N Csoka AB. Epigenetics across the human lifespan. Front Cell Dev Biol (2014) 2:49. doi: 10.3389/fcell.2014.00049 25364756 PMC4207041

[11] Condic ML. Totipotency: what it is and what it is not. Stem Cells Dev (2014) 23(8):796–812. doi: 10.1089/scd.2013.0364 24368070 PMC3991987

[12] Sun L Fu X Ma G Hutchins AP. Chromatin and epigenetic rearrangements in embryonic stem cell fate transitions. Front Cell Dev Biol (2021) 9:637309. doi: 10.3389/fcell.2021.637309 33681220 PMC7930395

[13] Okano M Bell DW Haber DA Li E. DNA Methyltransferases Dnmt3a and Dnmt3b are essential for de novo methylation and mammalian development. Cell (1999) 99(3):247–57. doi: 10.1016/S0092-8674(00)81656-6 10555141

[14] Avgustinova A Benitah SA. Epigenetic control of adult stem cell function. Nat Rev Mol Cell Biol (2016) 17(10):643–58. doi: 10.1038/nrm.2016.76 27405257

[15] Zoghbi HY Beaudet AL. Epigenetics and human disease. Cold Spring Harb Perspect Biol (2016) 8(2):a019497. doi: 10.1101/cshperspect.a019497 26834142 PMC4743078

[16] Soejima H Higashimoto K. Epigenetic and genetic alterations of the imprinting disorder beckwith-wiedemann syndrome and related disorders. J Hum Genet (2013) 58(7):402–9. doi: 10.1038/jhg.2013.51 23719190

[17] Ostrowski PJ Tatton-Brown K. Tatton-Brown-Rahman syndrome. In: Adam MP. editors. Seattle (WA): GeneReviews® [Internet] (1993). 35771960

[18] Shen W Heeley JM Carlston CM Acuna-Hidalgo R Nillesen WM Dent KM. The spectrum of DNMT3A variants in tatton-Brown-Rahman syndrome overlaps with that in hematologic malignancies. Am J Med Genet A (2017) 173(11):3022–8. doi: 10.1002/ajmg.a.38485 28941052

[19] Feinberg AP Vogelstein B. Hypomethylation distinguishes genes of some human cancers from their normal counterparts. Nature (1983) 301(5895):89–92. doi: 10.1038/301089a0 6185846

[20] Feinberg AP Tycko B. The history of cancer epigenetics. Nat Rev Cancer (2004) 4(2):143–53. doi: 10.1038/nrc1279 14732866

[21] Feinberg AP Koldobskiy MA Gondor A. Epigenetic modulators, modifiers and mediators in cancer aetiology and progression. Nat Rev Genet (2016) 17(5):284–99. doi: 10.1038/nrg.2016.13 PMC4888057 26972587

[22] Baylin SB Jones PA. Epigenetic determinants of cancer. Cold Spring Harb Perspect Biol (2016) 8(9). doi: 10.1101/cshperspect.a019505 PMC5008069 27194046

[23] Xie M Lu C Wang J McLellan MD Johnson KJ Wendl MC. Age-related mutations associated with clonal hematopoietic expansion and malignancies. Nat Med (2014) 20(12):1472–8. doi: 10.1038/nm.3733 PMC4313872 25326804

[24] Challen GA Goodell MA. Clonal hematopoiesis: mechanisms driving dominance of stem cell clones. Blood (2020) 136(14):1590–8. doi: 10.1182/blood.2020006510 PMC7530644 32746453

[25] Warren JT Link DC. Clonal hematopoiesis and risk for hematologic malignancy. Blood (2020) 136(14):1599–605. doi: 10.1182/blood.2019000991 PMC8209630 32736382

[26] Jaiswal S Ebert BL. Clonal hematopoiesis in human aging and disease. Science (2019) 366(6465):eaan4673. doi: 10.1126/science.aan4673 31672865 PMC8050831

[27] Gondek LP DeZern AE. Assessing clonal haematopoiesis: clinical burdens and benefits of diagnosing myelodysplastic syndrome precursor states. Lancet Haematol (2020) 7(1):e73–81. doi: 10.1016/S2352-3026(19)30211-X PMC7008978 31810765

[28] Audia JE Campbell RM. Histone modifications and cancer. Cold Spring Harb Perspect Biol (2016) 8(4):a019521. doi: 10.1101/cshperspect.a019521 27037415 PMC4817802

[29] Peng Y Croce CM. The role of MicroRNAs in human cancer. Signal Transduct Target Ther (2016) 1:15004. doi: 10.1038/sigtrans.2015.4 29263891 PMC5661652

[30] Sahraei M Chaube B Liu Y Sun J Kaplan A Price NL. Suppressing miR-21 activity in tumor-associated macrophages promotes an antitumor immune response. J Clin Invest (2019) 129(12):5518–36. doi: 10.1172/JCI127125 PMC6877327 31710308

[31] Feng YH Tsao CJ. Emerging role of microRNA-21 in cancer. BioMed Rep (2016) 5(4):395–402. doi: 10.3892/br.2016.747 27699004 PMC5038362

[32] Feng S De Carvalho DD. Clinical advances in targeting epigenetics for cancer therapy. FEBS J (2022) 289(5):1214–39. doi: 10.1111/febs.15750 33545740

[33] Liu Z Ren Y Weng S Xu H Li L Han X. A new trend in cancer treatment: the combination of epigenetics and immunotherapy. Front Immunol (2022) 13:809761. doi: 10.3389/fimmu.2022.809761 35140720 PMC8818678

[34] Srinageshwar B Maiti P Dunbar GL Rossignol J. Role of epigenetics in stem cell proliferation and differentiation: implications for treating neurodegenerative diseases. Int J Mol Sci (2016) 17(2):199. doi: 10.3390/ijms17020199 26848657 PMC4783933

[35] Cakouros D Gronthos S. Epigenetic regulation of bone marrow stem cell aging: revealing epigenetic signatures associated with hematopoietic and mesenchymal stem cell aging. Aging Dis (2019) 10(1):174–89. doi: 10.14336/AD.2017.1213 PMC6345334 30705777

[36] McClay JL Aberg KA Clark SL Nerella S Kumar G Xie LY. A methylome-wide study of aging using massively parallel sequencing of the methyl-CpG-enriched genomic fraction from blood in over 700 subjects. Hum Mol Genet (2014) 23(5):1175–85. doi: 10.1093/hmg/ddt511 PMC3919012 24135035

[37] Broske AM Vockentanz L Kharazi S Huska MR Mancini E Scheller M. DNA Methylation protects hematopoietic stem cell multipotency from myeloerythroid restriction. Nat Genet (2009) 41(11):1207–15. doi: 10.1038/ng.463 19801979

[38] Challen GA Sun D Jeong M Luo M Jelinek J Vasanthakumar A. Dnmt3a is essential for hematopoietic stem cell differentiation. Nat Genet (2011) 44(1):23–31. doi: 10.1182/blood.V118.21.386.386 22138693 PMC3637952

[39] Ko M Bandukwala HS An J Lamperti ED Thompson EC Hastie R. Ten-Eleven-Translocation 2 (TET2) negatively regulates homeostasis and differentiation of hematopoietic stem cells in mice. Proc Natl Acad Sci U.S.A. (2011) 108(35):14566–71. doi: 10.1073/pnas.1112317108 PMC3167529 21873190

[40] Moran-Crusio K Reavie L Shih A Abdel-Wahab O Ndiaye-Lobry D Lobry C. Tet2 loss leads to increased hematopoietic stem cell self-renewal and myeloid transformation. Cancer Cell (2011) 20(1):11–24. doi: 10.1016/j.ccr.2011.06.001 21723200 PMC3194039

[41] Challen GA Sun D Mayle A Jeong M Luo M Rodriguez B. Dnmt3a and Dnmt3b have overlapping and distinct functions in hematopoietic stem cells. Cell Stem Cell (2014) 15(3):350–64. doi: 10.1016/j.stem.2014.06.018 PMC4163922 25130491

[42] Jeong M Park HJ Celik H Ostrander EL Reyes JM Guzman A. Loss of Dnmt3a immortalizes hematopoietic stem cells *in vivo* . Cell Rep (2018) 23(1):1–10. doi: 10.1016/j.celrep.2018.03.025 29617651 PMC5908249

[43] Zhang CR Ostrander EL Kukhar O Mallaney C Sun J Haussler E. Txnip enhances fitness of Dnmt3a-mutant hematopoietic stem cells *via* p21. Blood Cancer Discov (2022) 3(3):220–39. doi: 10.1158/2643-3230.BCD-21-0132 PMC9414740 35394496

[44] Abdel-Wahab O Gao J Adli M Dey A Trimarchi T Chung YR. Deletion of Asxl1 results in myelodysplasia and severe developmental defects *in vivo* . J Exp Med (2013) 210(12):2641–59. doi: 10.1084/jem.20131141 PMC3832937 24218140

[45] Delhommeau F Dupont S Valle VD James C Trannoy S Massé A. Mutation in TET2 in myeloid cancers. N Engl J Med (2009) 360(22):2289–301. doi: 10.1056/NEJMoa0810069 19474426

[46] Genovese G Kähler AK Handsaker RE Lindberg J Rose SA Bakhoum SF. Clonal hematopoiesis and blood-cancer risk. N Engl J Med (2015) 372(11):1071–2. doi: 10.1056/NEJMoa1409405 25760361

[47] Jaiswal S. Clonal hematopoiesis and nonhematologic disorders. Blood (2020) 136(14):1606–14. doi: 10.1182/blood.2019000989 PMC8209629 32736379

[48] Medina EA Delma CR Yang FC. ASXL1/2 mutations and myeloid malignancies. J Hematol Oncol (2022) 15(1):127. doi: 10.1186/s13045-022-01336-x 36068610 PMC9450349

[49] Rodriguez RM Suarez-Alvarez B Salvanés R Muro M Martínez-Camblor P Colado E. DNA Methylation dynamics in blood after hematopoietic cell transplant. PloS One (2013) 8(2):e56931. doi: 10.1371/journal.pone.0056931 23451113 PMC3579934

[50] Wahlestedt M Norddahl GL Sten G Ugale A Frisk M-AM Mattsson R. An epigenetic component of hematopoietic stem cell aging amenable to reprogramming into a young state. Blood (2013) 121(21):4257–64. doi: 10.1182/blood-2012-11-469080 23476050

[51] Weidner CI Ziegler P Hahn M Brümmendorf TH Ho AD Dreger P. Epigenetic aging upon allogeneic transplantation: the hematopoietic niche does not affect age-associated DNA methylation. Leukemia (2015) 29(4):985–8. doi: 10.1038/leu.2014.323 25388956

[52] Søraas A Matsuyama M de Lima M Wald D Buechner J Gedde-Dahl T. Epigenetic age is a cell-intrinsic property in transplanted human hematopoietic cells. Aging Cell (2019) 18(2):e12897. doi: 10.1111/acel.12897 30712319 PMC6413751

[53] Stölzel F Brosch M Horvath S Kramer M Thiede C von Bonin M. Dynamics of epigenetic age following hematopoietic stem cell transplantation. Haematologica (2017) 102(8):e321–3. doi: 10.3324/haematol.2016.160481 PMC5541887 28550187

[54] Alsaggaf R Katta S Wang T Hicks BD Zhu B Spellman SR. Epigenetic aging and hematopoietic cell transplantation in patients with severe aplastic anemia. Transplant Cell Ther (2021) 27(4):313.e1–8. doi: 10.1016/j.jtct.2021.01.013 PMC8036238 33836872

[55] Frobel J Rahmig S Franzen J Waskow C Wagner W. Epigenetic aging of human hematopoietic cells is not accelerated upon transplantation into mice. Clin Epigenet (2018) 10:67. doi: 10.1186/s13148-018-0499-7 PMC5964682 29796118

[56] Frick M Chan W Arends CM Hablesreiter R Halik A Heuser M. Role of donor clonal hematopoiesis in allogeneic hematopoietic stem-cell transplantation. J Clin Oncol (2019) 37(5):375–85. doi: 10.1200/JCO.2018.79.2184 30403573

[57] Christopher J Gibson M. (2020). DNMT3A clonal hematopoiesis in older donors is associated with improved survival in recipients after allogeneic hematopoietic cell transplant, in: ASH: 62nd ASH Annual Meeting.

[58] Gibson CJ Kim HT Zhao L Murdock HM Hambley B Ogata A. Donor clonal hematopoiesis and recipient outcomes after transplantation. J Clin Oncol (2022) 40(2):189–201. doi: 10.1200/JCO.21.02286 34793200 PMC8718176

[59] Wachsmuth LP Patterson MT Eckhaus MA Venzon DJ Gress RE Kanakry CG. Post-transplantation cyclophosphamide prevents graft-versus-host disease by inducing alloreactive T cell dysfunction and suppression. J Clin Invest (2019) 129(6):2357–73. doi: 10.1172/JCI124218 PMC6546453 30913039

[60] Burns SS Kapur R. Clonal hematopoiesis of indeterminate potential as a novel risk factor for donor-derived leukemia. Stem Cell Rep (2020) 15(2):279–91. doi: 10.1016/j.stemcr.2020.07.008 PMC7419737 32783925

[61] Oran B Champlin RE Wang F Tanaka T Saliba RM Al-Atrash G. Donor clonal hematopoiesis increases risk of acute graft versus host disease after matched sibling transplantation. Leukemia (2021) 36:298. doi: 10.1038/s41375-021-01430-y 34876697

[62] Kim KH Kim T Novitzky-Basso I Lee H Yoo Y Ahn J-S. Clonal hematopoiesis in the donor does not adversely affect long-term outcomes following allogeneic hematopoietic stem cell transplantation: result from 13-year followup. Haematologica (2023). doi: 10.3324/haematol.2022.281806 PMC10316278 36727396

[63] Newell LF Dunlap J Gatter K Bagby GC Press RD Cook RJ. Graft-versus-host disease after liver transplantation is associated with bone marrow failure, hemophagocytosis, and DNMT3A mutations. Am J Transplant (2021) 21(12):3894–906. doi: 10.1111/ajt.16635 33961341

[64] Ktena YP Koldobskiy MA Barbato MI Fu H-H Luznik L Llosa NJ. Donor T cell DNMT3a regulates alloreactivity in mouse models of hematopoietic stem cell transplantation. J Clin Invest (2022) 132(13):e158047. doi: 10.1172/JCI158047 35608905 PMC9246380

[65] West EE Youngblood B Tan WG Jin H-T Araki K Alexe G. Tight regulation of memory CD8(+) T cells limits their effectiveness during sustained high viral load. Immunity (2011) 35(2):285–98. doi: 10.1016/j.immuni.2011.05.017 PMC3241982 21856186

[66] Thomas RM Gamper CJ Ladle BH Powell JD Wells AD. De novo DNA methylation is required to restrict T helper lineage plasticity. J Biol Chem (2012) 287(27):22900–9. doi: 10.1074/jbc.M111.312785 PMC3391093 22584578

[67] Ladle BH Li K-P Phillips MJ Pucsek AB Haile A Powell JD. De novo DNA methylation by DNA methyltransferase 3a controls early effector CD8+ T-cell fate decisions following activation. Proc Natl Acad Sci USA (2016) 113(38):10631–6. doi: 10.1073/pnas.1524490113 PMC5035851 27582468

[68] Bird JJ Brown DR Mullen AC Moskowitz NH Mahowald MA Sider JR. Helper T cell differentiation is controlled by the cell cycle. Immunity (1998) 9(2):229–37. doi: 10.1016/S1074-7613(00)80605-6 9729043

[69] Gamper CJ Agoston AT Nelson WG Powell JD. Identification of DNA methyltransferase 3a as a T cell receptor-induced regulator of Th1 and Th2 differentiation. J Immunol (2009) 183(4):2267–76. doi: 10.4049/jimmunol.0802960 PMC2818975 19625655

[70] Lee GR Kim ST Spilianakis CG Fields PE Flavell RA. T Helper cell differentiation: regulation by cis elements and epigenetics. Immunity (2006) 24(4):369–79. doi: 10.1016/j.immuni.2006.03.007 16618596

[71] Ghoneim HE Fan Y Moustaki A Abdelsamed HA Dash P Dogra P. De Novo epigenetic programs inhibit PD-1 blockade-mediated T cell rejuvenation. Cell (2017) 170(1):142–157.e19. doi: 10.1016/j.cell.2017.06.007 28648661 PMC5568784

[72] Prinzing B Zebley CC Petersen CT Fan Y Anido AA Yi Z. Deleting DNMT3A in CAR T cells prevents exhaustion and enhances antitumor activity. Sci Transl Med (2021) 13(620):eabh0272. doi: 10.1126/scitranslmed.abh0272 34788079 PMC8733956

[73] Zebley CC Brown C Mi T Fan Y Alli S Boi S. CD19-CAR T cells undergo exhaustion DNA methylation programming in patients with acute lymphoblastic leukemia. Cell Rep (2021) 37(9):110079. doi: 10.1016/j.celrep.2021.110079 34852226 PMC8800370

[74] Lubbert M Bertz H Rüter B Marks R Claus R Wäsch R. Non-intensive treatment with low-dose 5-aza-2’-deoxycytidine (DAC) prior to allogeneic blood SCT of older MDS/AML patients. Bone Marrow Transplant (2009) 44(9):585–8. doi: 10.1038/bmt.2009.64 19363531

[75] Cogle CR Imanirad I Wiggins LE Hsu J Brown R Scornik JC. Hypomethylating agent induction therapy followed by hematopoietic cell transplantation is feasible in patients with myelodysplastic syndromes. Clin Adv Hematol Oncol (2010) 8(1):40–6. 20351682

[76] Gerds AT Gooley TA Estey EH Appelbaum FR Deeg HJ Scott BL. Pretransplantation therapy with azacitidine vs induction chemotherapy and posttransplantation outcome in patients with MDS. Biol Blood Marrow Transplant (2012) 18(8):1211–8. doi: 10.1016/j.bbmt.2012.01.009 PMC3376402 22252125

[77] Mohty R El Hamed R Brissot E Bazarbachi A Mohty M. New drugs before, during, and after hematopoietic stem cell transplantation for patients with acute myeloid leukemia. Haematologica (2023) 108(2):321–41. doi: 10.3324/haematol.2022.280798 PMC9890036 36722403

[78] Nayak RK Chen YB. Maintenance therapy for AML after allogeneic HCT. Front Oncol (2022) 12:895771. doi: 10.3389/fonc.2022.895771 36016625 PMC9397403

[79] Yan Y Upadhyaya R Zhang VW Berg T. Epigenetic maintenance strategies after allogeneic stem cell transplantation in acute myeloid leukemia. Exp Hematol (2022) 109:1–10 e1. doi: 10.1016/j.exphem.2022.03.003 35288231

[80] Choi J Ritchey J Prior JL Holt M Shannon WD Deych E. *In vivo* administration of hypomethylating agents mitigate graft-versus-host disease without sacrificing graft-versus-leukemia. Blood (2010) 116(1):129–39. doi: 10.1182/blood-2009-12-257253 PMC2904576 20424188

[81] Goodyear OC Dennis M Jilani NY Loke J Siddique S Ryan G. Azacitidine augments expansion of regulatory T cells after allogeneic stem cell transplantation in patients with acute myeloid leukemia (AML). Blood (2012) 119(14):3361–9. doi: 10.1182/blood-2011-09-377044 22234690

[82] Cooper ML Choi J Karpova D Vij K Ritchey J Schroeder MA. Azacitidine mitigates graft-versus-Host disease *via* differential effects on the proliferation of T effectors and natural regulatory T cells *in vivo* . J Immunol (2017) 198(9):3746–54. doi: 10.4049/jimmunol.1502399 PMC5541679 28330901

[83] Oran B de Lima M Garcia-Manero G Thall PF Lin R Popat U. A phase 3 randomized study of 5-azacitidine maintenance vs observation after transplant in high-risk AML and MDS patients. Blood Adv (2020) 4(21):5580–8. doi: 10.1182/bloodadvances.2020002544 PMC7656915 33170934

[84] Gao L Zhang Y Wang S Kong P Su Y Hu J. Effect of rhG-CSF combined with decitabine prophylaxis on relapse of patients with high-risk MRD-negative AML after HSCT: an open-label, multicenter, randomized controlled trial. J Clin Oncol (2020) 38(36):4249–59. doi: 10.1200/JCO.19.03277 PMC7768335 33108244

[85] Wei Y Xiong X Li X Lu W He X Jin X. Low-dose decitabine plus venetoclax is safe and effective as post-transplant maintenance therapy for high-risk acute myeloid leukemia and myelodysplastic syndrome. Cancer Sci (2021) 112(9):3636–44. doi: 10.1111/cas.15048 PMC8409404 34185931

[86] Mehdipour P Chen R De Carvalho DD. The next generation of DNMT inhibitors. Nat Cancer (2021) 2(10):1000–1. doi: 10.1038/s43018-021-00271-z 35121882

[87] Li A Abraham C Wang Y Zhang Y. New insights into the basic biology of acute graft-versus-host-disease. Haematologica (2020) 105(11):2540–9. doi: 10.3324/haematol.2019.240291 PMC7604569 33131244

[88] Kim S Santhanam S Lim S Choi J. Targeting histone deacetylases to modulate graft-Versus-Host disease and graft-Versus-Leukemia. Int J Mol Sci (2020) 21(12):4281. doi: 10.3390/ijms21124281 32560120 PMC7349873

[89] Reddy P Maeda Y Hotary K Liu C Reznikov LL Dinarello CA. Histone deacetylase inhibitor suberoylanilide hydroxamic acid reduces acute graft-versus-host disease and preserves graft-versus-leukemia effect. Proc Natl Acad Sci U.S.A. (2004) 101(11):3921–6. doi: 10.1073/pnas.0400380101 PMC374345 15001702

[90] Reddy P Sun Y Toubai T Duran-Struuck R Clouthier SG Weisiger E. Histone deacetylase inhibition modulates indoleamine 2,3-dioxygenase-dependent DC functions and regulates experimental graft-versus-host disease in mice. J Clin Invest (2008) 118(7):2562–73. doi: 10.1172/JCI34712 PMC2430497 18568076

[91] Choi SW Gatza E Hou G Sun Y Whitfield J Song Y. Histone deacetylase inhibition regulates inflammation and enhances tregs after allogeneic hematopoietic cell transplantation in humans. Blood (2015) 125(5):815–9. doi: 10.1182/blood-2014-10-605238 PMC4311228 25428224

[92] Wang L Beier UH Akimova T Dahiya S Han R Samanta A. Histone/protein deacetylase inhibitor therapy for enhancement of Foxp3+ T-regulatory cell function posttransplantation. Am J Transplant (2018) 18(7):1596–603. doi: 10.1111/ajt.14749 PMC6035084 29603600

[93] Choi SW Braun T Chang L Ferrara JLM Pawarode A Magenau JM. Vorinostat plus tacrolimus and mycophenolate to prevent graft-versus-host disease after related-donor reduced-intensity conditioning allogeneic haemopoietic stem-cell transplantation: a phase 1/2 trial. Lancet Oncol (2014) 15(1):87–95. doi: 10.1016/S1470-2045(13)70512-6 24295572 PMC4103793

[94] Choi SW Braun T Henig I Gatza E Magenau J Parkin B. Vorinostat plus tacrolimus/methotrexate to prevent GVHD after myeloablative conditioning, unrelated donor HCT. Blood (2017) 130(15):1760–7. doi: 10.1182/blood-2017-06-790469 PMC5639486 28784598

[95] Perez L Fernandez H Horna P Riches M Locke F Field T. Phase I trial of histone deacetylase inhibitor panobinostat in addition to glucocorticoids for primary therapy of acute graft-versus-host disease. Bone Marrow Transplant (2018) 53(11):1434–44. doi: 10.1038/s41409-018-0163-z PMC7771280 29670210

[96] Perez L Fernandez H Kharfan-Dabaja M Khimani F Betts B Mishra A. A phase 2 trial of the histone deacetylase inhibitor panobinostat for graft-versus-host disease prevention. Blood Adv (2021) 5(13):2740–50. doi: 10.1182/bloodadvances.2021004225 PMC8288668 34242388

[97] He S Wang J Kato K Xie F Varambally S Mineishi S. Inhibition of histone methylation arrests ongoing graft-versus-host disease in mice by selectively inducing apoptosis of alloreactive effector T cells. Blood (2012) 119(5):1274–82. doi: 10.1182/blood-2011-06-364422 PMC3338164 22117046

[98] He S Xie F Liu Y Tong Q Mochizuki K Lapinski PE. The histone methyltransferase Ezh2 is a crucial epigenetic regulator of allogeneic T-cell responses mediating graft-versus-host disease. Blood (2013) 122(25):4119–28. doi: 10.1182/blood-2013-05-505180 PMC3862282 24141370

[99] Alahmari B Cooper M Ziga E Ritchey J DiPersio JF Choi J. Selective targeting of histone modification fails to prevent graft versus host disease after hematopoietic cell transplantation. PloS One (2018) 13(11):e0207609. doi: 10.1371/journal.pone.0207609 30452487 PMC6242356

[100] Doroshow DB Eder JP LoRusso PM. BET inhibitors: a novel epigenetic approach. Ann Oncol (2017) 28(8):1776–87. doi: 10.1093/annonc/mdx157 28838216

[101] Sun Y Wang Y Toubai T Oravecz-Wilson K Liu C Mathewson N. BET bromodomain inhibition suppresses graft-versus-host disease after allogeneic bone marrow transplantation in mice. Blood (2015) 125(17):2724–8. doi: 10.1182/blood-2014-08-598037 PMC4408296 25778533

[102] Copsel SN Lightbourn CO Barreras H Lohse I Wolf D Bader CS. BET bromodomain inhibitors which permit treg function enable a combinatorial strategy to suppress GVHD in pre-clinical allogeneic HSCT. Front Immunol (2018) 9:3104. doi: 10.3389/fimmu.2018.03104 30733722 PMC6353853

[103] Snyder KJ Choe HK Gao Y Sell NE Braunreiter KM Zitzer NC. Inhibition of bromodomain and extra terminal (BET) domain activity modulates the IL-23R/IL-17 axis and suppresses acute graft-Versus-Host disease. Front Oncol (2021) 11:760789. doi: 10.3389/fonc.2021.760789 34722316 PMC8554203

[104] Zaiken MC Flynn R Paz KG Rhee SY Jin S Mohamed FA. BET-bromodomain and EZH2 inhibitor-treated chronic GVHD mice have blunted germinal centers with distinct transcriptomes. Blood (2022) 139(19):2983–97. doi: 10.1182/blood.2021014557 PMC9101246 35226736

[105] Peltier D Reddy P. Non-coding RNA mediated regulation of allogeneic T cell responses after hematopoietic transplantation. Front Immunol (2018) 9:1110. doi: 10.3389/fimmu.2018.01110 29963039 PMC6013767

[106] Vajari MK Moradinasab S Yousefi A-M Bashash D. Noncoding RNAs in diagnosis and prognosis of graft-versus-host disease (GVHD). J Cell Physiol (2022) 237(9):3480–95. doi: 10.1002/jcp.30830 35842836

[107] Koenecke C Krueger A. MicroRNA in T-cell development and T-cell mediated acute graft-Versus-Host disease. Front Immunol (2018) 9:992. doi: 10.3389/fimmu.2018.00992 29867969 PMC5949326

[108] Wu Y Mealer C Schutt S Wilson CL Bastian D Sofi MH. MicroRNA-31 regulates T-cell metabolism *via* HIF1alpha and promotes chronic GVHD pathogenesis in mice. Blood Adv (2022) 6(10):3036–52. doi: 10.1182/bloodadvances.2021005103 PMC9131913 35073581

[109] Ranganathan P Heaphy CEA Costinean S Stauffer N Na C Hamadani M. Regulation of acute graft-versus-host disease by microRNA-155. Blood (2012) 119(20):4786–97. doi: 10.1182/blood-2011-10-387522 PMC3367879 22408260

[110] Chen S Smith BAH Iype J Prestipino A Pfeifer D Grundmann S. MicroRNA-155-deficient dendritic cells cause less severe GVHD through reduced migration and defective inflammasome activation. Blood (2015) 126(1):103–12. doi: 10.1182/blood-2014-12-617258 25972159

[111] Wu Y Heinrichs J Bastian D Fu J Nguyen H Schutt S. MicroRNA-17-92 controls T-cell responses in graft-versus-host disease and leukemia relapse in mice. Blood (2015) 126(11):1314–23. doi: 10.1182/blood-2015-02-627356 PMC4566810 26138686

[112] Stickel N Prinz G Pfeifer D Hasselblatt P Schmitt-Graeff A Follo M. MiR-146a regulates the TRAF6/TNF-axis in donor T cells during GVHD. Blood (2014) 124(16):2586–95. doi: 10.1182/blood-2014-04-569046 25205119

[113] Gartner JG Durston MM Booth SA Ellison CA. Systemic treatment with a miR-146a mimic suppresses endotoxin sensitivity and partially protects mice from the progression of acute graft-versus-Host disease. Scand J Immunol (2017) 86(5):368–76. doi: 10.1111/sji.12597 28853768

[114] Stickel N Hanke K Marschner D Prinz G Köhler M Melchinger W. MicroRNA-146a reduces MHC-II expression *via* targeting JAK/STAT signaling in dendritic cells after stem cell transplantation. Leukemia (2017) 31(12):2732–41. doi: 10.1038/leu.2017.137 PMC6231537 28484267

[115] Atarod S Norden J Bibby LA Janin A Ratajczak P Lendrem C. Differential MicroRNA expression levels in cutaneous acute graft-Versus-Host disease. Front Immunol (2018) 9:1485. doi: 10.3389/fimmu.2018.01485 30042760 PMC6048189

[116] Lacina P Crossland RE Wielińska J Czyż A Szeremet A Ussowicz M. Differential expression of miRNAs from extracellular vesicles in chronic graft-versus-host disease: a preliminary study. Adv Clin Exp Med (2022). doi: 10.17219/acem/155373 36398373

[117] Peltier D Radosevich M Ravikumar V Pitchiaya S Decoville T Wood SC. RNA-Seq of human T cells after hematopoietic stem cell transplantation identifies Linc00402 as a regulator of T cell alloimmunity. Sci Transl Med (2021) 13(585):eaaz0316. doi: 10.1126/scitranslmed.aaz0316 33731431 PMC8589011

[118] Peltier DC Roberts A Reddy P. LNCing RNA to immunity. Trends Immunol (2022) 43(6):478–95. doi: 10.1016/j.it.2022.04.002 PMC9647660 35501219

[119] Wang F Luo L Gu Z Yang N Wang L Gao C. Integrative analysis of long noncoding RNAs in patients with graft-versus-Host disease. Acta Haematol (2020) 143(6):533–51. doi: 10.1159/000505255 32289782

[120] Zhao F Zhang X Pei X Yang D Han M. Deregulated expression of circular RNAs is associated with immune evasion and leukemia relapse after allogeneic hematopoietic stem cell transplantation. Genes (Basel) (2022) 13(11):1986. doi: 10.3390/genes13111986 36360223 PMC9689715

